# Inhibition of endoplasmic reticulum (ER) stress sensors sensitizes cancer stem-like cells to ER stress-mediated apoptosis

**DOI:** 10.18632/oncotarget.10126

**Published:** 2016-06-17

**Authors:** Asaha Fujimoto, Kei Kawana, Ayumi Taguchi, Katsuyuki Adachi, Masakazu Sato, Hiroe Nakamura, Juri Ogishima, Mitsuyo Yoshida, Tomoko Inoue, Haruka Nishida, Kensuke Tomio, Aki Yamashita, Yoko Matsumoto, Takahide Arimoto, Osamu Wada-Hiraike, Katsutoshi Oda, Takeshi Nagamatsu, Yutaka Osuga, Tomoyuki Fujii

**Affiliations:** ^1^ Department of Obstetrics and Gynecology, Graduate School of Medicine, The University of Tokyo, Tokyo 113-8655, Japan

**Keywords:** cancer stem-like cell, sphere forming cell, endoplasmic reticulum (ER) stress, cisplatin, tunicamycin

## Abstract

Although cancer stem cells (CSC) have been implicated in the development of resistance to anti-cancer therapy including chemotherapy, the mechanisms underlying chemo-resistance by CSC have not yet been elucidated. We herein isolated sphere-forming (cancer stem-like) cells from the cervical cancer cell line, SiHa, and examined the unfolded protein reaction (UPR) to chemotherapeutic-induced endoplasmic reticulum (ER) stress. We revealed that tunicamycin-induced ER stress-mediated apoptosis occurred in monolayer, but not sphere-forming cells. Biochemical assays demonstrated that sphere-forming cells were shifted to pro-survival signaling through the inactivation of IRE1 (XBP-1 splicing) and activation of PERK (elF2α phosphorylation) branches under tunicamycin-induced ER stress conditions. The proportion of apoptotic cells among sphere-forming cells was markedly increased by the tunicamycin+PERK inhibitor (PERKi) treatment, indicating that PERKi sensitized sphere-forming cells to tunicamycin-induced apoptosis. Cisplatin is also known to induce ER stress-mediated apoptosis. A low concentration of cisplatin failed to shift sphere-forming cells to apoptosis, although IRE1 branch, but not PERK, was activated. ER stress-mediated apoptosis occurred in sphere-forming cells by the cisplatin+IRE1α inhibitor (IRE1i) treatment. IRE1i, synergistic with cisplatin, up-regulated elF2α phosphorylation, and this was followed by the induction of CHOP in sphere-forming cells. The results of the present study demonstrated that the inhibition of ER stress sensors, combined with ER stress-inducible chemotherapy, shifted cancer stem-like cells to ER stress-mediated apoptosis.

## INTRODUCTION

Although cancer stem cells (CSC) account for only a small population of disseminated tumor cells, they exhibit several unique properties that are similar to those of adult stem cells, but are not observed in the majority of cancer cells. One of the most important properties of CSC is the ability to self-renew, by which CSC are not only a progenitor of cancer cells, but also maintain the population of CSC [1–3]. CSC have also been implicated in the development of resistance to chemotherapy, which may result in the recurrence or metastasis of disseminated cancer cells [4, 5]. Previous studies revealed a relationship between CSC features and aggressive cancer dissemination which is resulted from chemo-resistance and metastasis [6–8].

Anti-apoptotic mechanisms have been suggested to involve the immortalization and chemo-resistance of malignant cells. A transcriptome analysis of ovarian cancer recently revealed that carboplatin-resistant high-grade serous ovarian cancer (HGSC) showed the up-regulation of anti-apoptotic signals as well as DNA repair signals [9]. The clinical samples examined in that study were obtained from CA125-negative HGSC cells, which are poorly differentiated and have similar features to CSC. A number of anti-apoptotic systems in cancer have been examined in detail [10–12] and the blockade of these systems may represent a successful strategy for cancer therapy including that for chemo-resistant tumors. Therefore, apoptosis resistance in CSC may be a crucial mechanism for chemo-resistance in disseminated tumors. However, anti-apoptotic mechanisms have not yet been elucidated in CSC.

The endoplasmic reticulum (ER) is a multi-function organelle that supports ubiquitous cellular metabolic processes including protein synthesis, post-translational modifications to and the translocation of glycoproteins, the protein quality control checking, and calcium ion homeostasis. ER stress, including endogenous stress, such as the accumulation of disturbed glycoproteins, or exogenous stress, such as chemotherapeutics and hypoxia, activate cellular responses to stress, termed the unfolded protein response (UPR). UPR plays a crucial role in the maintenance of ER homeostasis. Three major UPR branches and proximal sensors have been identified: PKR-like ER stress kinase (PERK), inositol-requiring transmembrane kinase and endonuclease 1 (IRE1), and activating transcription factor 6 (ATF6) [13]. PERK phosphorylates elF2α while IRE1 induces the splicing of X-box binding protein 1 (XBP-1) and activates the TRAF2/JNK pathway. ATF6 cleaved at the Golgi apparatus up-regulates UPR genes. Three ER stress sensors are inactivated by binding to the binding immunoglobulin protein (BiP) and/or unfolded proteins under steady conditions. Once ER stress occurs, the BiP/unfolded protein complex dissociates from ER stress sensors. This accompanies the activation of their signaling pathways and consequently blocks the initiation of protein synthesis until the cell recovers from ER stress, namely, the cell shifts to pro-survival [14].

Prolonged ER stress has been shown to induce ER stress-mediated apoptosis in order to clear cells with disrupted ER functions [15]. Phosphorylated elF2α (p-elF2α) and spliced XBP-1 up-regulate the expression of UPR genes (including GRP78) and the C/EBP-homologous protein (CHOP). CHOP leads to the induction of genes involved in cell death and the suppression of p-elF2α which is a major branch involved in cell quiescence via the translational repression of cell survival [15]. IRE1 also promotes the caspase pathway through the activation of c-Jun N-terminal kinase (JNK). Therefore, UPR in response to ER stress has opposing effects: pro-apoptotic and pro-survival (anti-apoptotic) signaling. Cellular fates are determined by the timing of PERK and IRE1 signaling in a complex manner as well as in a cell type-dependent manner [15–17].

Cervical cancer is known to be caused by oncogenic human papillomavirus (HPV) and to ubiquitously express viral oncogenes E6 and E7 at high levels [18]. E6 and E6AP synergistically degrade p53 via a proteasome pathway and E6 also activates hTERT [19, 20]. These findings imply that p53-dependent apoptosis may be ignored in sphere-forming and monolayer cells derived from SiHa cells. SiHa cells appeared to be suitable for examining ER stress-mediated apoptosis.

Based on the importance of CSC in chemo-resistance and opposing cellular fates in the presence of ER stress in a cell-specific manner, we herein investigate UPR in response to ER stress including chemotherapeutics in CSC. We demonstrated that cancer stem-like (sphere-forming) cells derived from SiHa cells resisted ER stress-mediated apoptosis and that signaling activated with chemotherapeutic ER stress was different between sphere-forming and monolayer cells. The combination of cisplatin and an IRE1α inhibitor had a synergistic effect on ER stress-mediated apoptosis. Our results demonstrated that the inhibition of ER stress sensors combined with chemotherapy has potential as a novel therapeutic strategy.

## RESULTS AND DISCUSSION

### Isolation and characterization of cancer stem-like cells derived from a cervical cancer cell line

**Figure 1:**
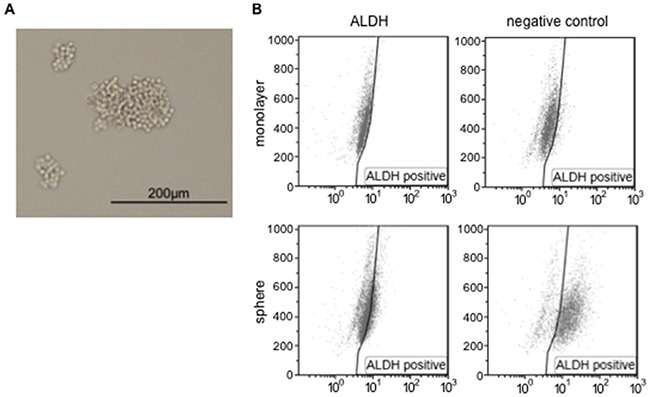
Sphere-forming cells were isolated from SiHa cells **A.** A representative microscopic image of SiHa-derived sphere-forming cells. The scale bar indicates 200 μm. **B.** Monolayer or sphere-forming cells were treated with Aldefluor reagent alone (left) or in the presence of the ALDH inhibitor DEAB (negative control, right), and were analyzed by flow cytometry.

Previous studies have used cancer sphere-forming cells isolated from various malignant cell lines and clinical samples to investigate the properties of CSC [21, 22]. We herein isolated sphere-forming cells derived from the cervical cancer cell line, SiHa (HPV16-positive squamous cell carcinoma), according to the standard protocol for sphere formation using a low-attachment plate and serum-free media [23]. A representative microscopic picture showed that several sphere-forming, but not monolayer cells were observed on the low-attachment plate (Figure 1A). In order to confirm whether sphere-forming cells possess the features of cancer stem-like cells, we investigated cell populations exhibiting high ALDH enzymatic activity among monolayer or sphere-forming cells. Most (78.5%) sphere-forming cells were positive for high ALDH activity, in contrast to only 13.9% of monolayer cells (Figure 1B), indicating that the sphere-forming cells we collected were cervical cancer stem-like cells that possess the features of CSC. Some basic and clinical studies on cervical cancer reported that the CSC or cancer stem-like cells of cervical cancer expressed ALDH, CD44, and CD49f [7, 21]. ALDH was strongly expressed in sphere-forming cells in the present study, while the expression of CD44 and CD49f was slightly stronger than that in monolayer cells (Supplementary Figure S1). Monolayer cells have been suggested to include a small population of sphere-forming cells; however, since they were in the minority, monolayer cells showed different features from those of sphere-forming cells.

### ER stress-mediated apoptosis did not occur in cancer stem-like cells

**Figure 2:**
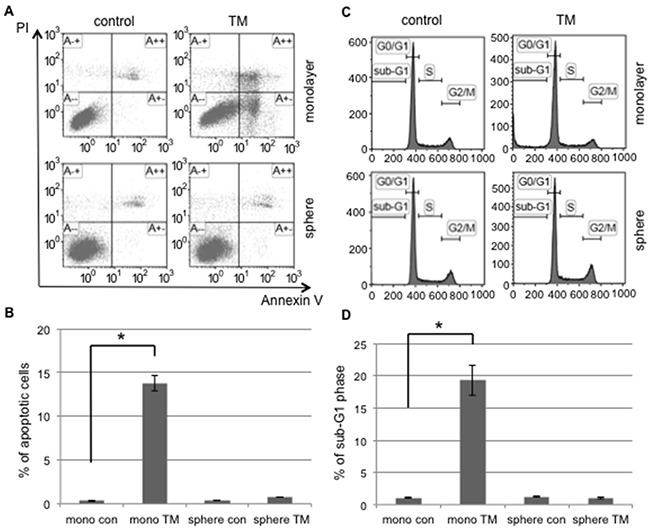
Tunicamycin-induced apoptosis occurred in monolayer cells, but not in sphere-forming cells **A.** Monolayer (mono) or sphere-forming (sphere) cells were untreated (control: con, left) or treated with 0.03 μM tunicamycin (TM, right) for 72 hours, and then subjected to PI/Annexin-V staining and analyzed by flow cytometry. **B.** A quantitative analysis of PI-negative/Annexin-V-positive apoptotic cells showed that tunicamycin-induced apoptosis only occurred in monolayer cells. The values shown represent the means ± SEM (*p < 0.05). **C.** Monolayer or sphere-forming cells were untreated (left) or treated with 0.03 μM tunicamycin (right) for 72 hours, then fixed and stained with propidium iodide for a flow cytometry assay. **D.** A quantitative analysis of sub-G1 region (M1) cells showed that tunicamycin-induced apoptosis only occurred in monolayer cells. The values shown represent the means ± SEM (*p < 0.05).

In the tumor microenvironment, exogenous and prolonged ER stress, such as that caused by chemotherapy and radiotherapy, induces ER stress-mediated apoptosis in order to clear cells with disrupted ER functions. We investigated the susceptibility of cancer cells to apoptosis induced by ER stress in monolayer and sphere-forming cells. Monolayer or sphere-forming cells were exposed to tunicamycin, an ER stress inducer, and apoptotic cells were counted using flow cytometry for PI/Annexin-V (Figure 2). Apoptotic cells accounted for only 0.7% of sphere-forming cells and 13.8% of monolayer cells (Figure 2A). A flow cytometric analysis showed that tunicamycin-induced apoptosis occurred in monolayer cells, but not in sphere-forming cells (Figure 2B). Furthermore, a flow cytometric analysis on cell cycle proportions demonstrated that the sub-G1 population was clearly increased in monolayer cells exposed to tunicamycin, but not in sphere-forming cells (Figure 2C and 2D). These results suggest that cellular fates in response to tunicamycin-mediated ER stress differed between monolayer and sphere-forming cells, with the latter being resistant to tunicamycin-induced apoptosis.

### Differences in UPR to ER stress sensors between cancer stem-like and cancer cells

**Figure 3:**
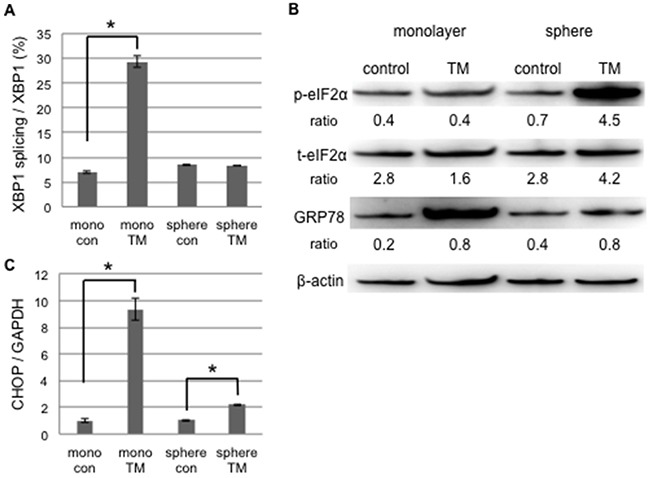
UPR to tunicamycin-induced ER stress differed between cancer stem-like and cancer cells Monolayer (mono) or sphere-forming (sphere) cells were untreated (control: con) or treated with 0.03 μM tunicamycin (TM) for 72 hours, and live cells were fractioned. **A.** Total RNA was extracted for RT-PCR and the ratio of spliced XBP1 mRNA to total XBP1 mRNA was calculated using the comparative Ct method. XBP1 splicing was increased by tunicamycin in monolayer cells. The values shown represent the means ± SEM (*p < 0.05). **B.** Cell extracts were prepared for Western blotting of the indicated proteins, and representative blots are shown. The ratio indicated that each protein level was normalized by β-actin levels (loading control). The phosphorylation of eIF2α was increased by tunicamycin in sphere-forming cells. **C.** The relative expression of CHOP was calculated and normalized by GAPDH. The values shown represent the means ± SEM (*p < 0.05).

We examined the difference in UPR to tunicamycin-mediated ER stress between monolayer and sphere-forming cells, with a focus on pro- and anti-apoptotic ER stress-mediated pathways. We assessed the splicing of XBP-1 and phosphorylation of elF2α by RT-qPCR and Western blotting, respectively (Figure 3A and 3B). XBP-1 splicing was clearly increased by tunicamycin in monolayer cells, but was absent in sphere-forming cells (Figure 3A). Western blotting for elF2α and a semi-quantitative analysis of the bands showed that the ratio of p-elF2α/β-actin was increased 6.4-fold in sphere-forming cells, while it was not increased in monolayer cells by tunicamycin (Figure 3B). The expression of CHOP and GRP78 was clearly increased by tunicamycin in monolayer cells, but only negligibly so in sphere-forming cells (Figure 3B and 3C). In monolayer cells, ER homeostasis was disrupted during the tunicamycin treatment. Our results indicate that the UPR balance shifted to pro-apoptotic signaling through the preferential activation of the IRE1 branch followed by CHOP-mediated apoptosis and also through the suppression of the PERK/p-elF2α branch by the increased expression of CHOP, which blocked pro-survival signaling by the PERK branch. In contrast, in sphere-forming cells, the PERK branch was preferentially activated and elF2α was then strongly phosphorylated by the tunicamycin treatment, suggesting that UPR shifted to pro-survival signaling. The lack of XBP-1 splicing indicated that the IRE1 branch did not play a crucial role in tunicamycin-induced ER stress in sphere-forming cells. The slight increase observed in the expression of CHOP and GRP78 was attributed to p-elF2α/ATF4 and/or ATF6. Sphere-forming cells had the ability to shift from pro-apoptotic to pro-survival signaling through the inactivation of the IRE1 branch and activation of the PERK branch, at least under tunicamycin-induced ER stress.

### Inhibitors of ER stress sensors induced ER stress-mediated apoptosis in cancer stem-like cells

**Figure 4:**
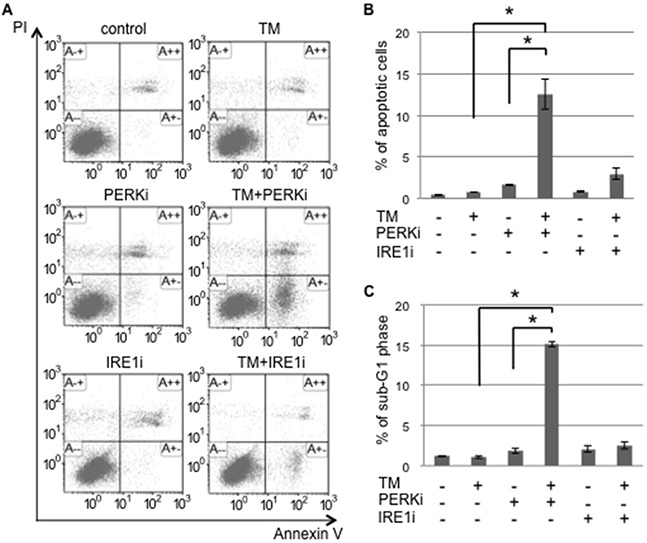
The PERK inhibitor induced tunicamycin-mediated apoptosis in cancer stem-like cells Sphere-forming cells were untreated (control) or treated with 0.03 μM tunicamycin (TM) and/or 1.5 μM GSK 2606414 (a PERK inhibitor: PERKi), or 7 μM 4μ8C (an IRE1α inhibitor: IRE1i) for 72 hours. **A.** After the treatment, cells were subjected to PI/Annexin-V staining and analyzed by flow cytometry. **B.** A quantitative analysis of PI-negative/Annexin-V-positive apoptotic cells showed that the proportion of apoptotic cells in sphere-forming cells was clearly increased by the PERKi combined with tunicamycin treatment. The values shown represent the means ± SEM (*p < 0.05). **C.** After being treated, cells were fixed and stained with propidium iodide for a flow cytometry assay. A quantitative analysis of sub-G1 region (M1) cells showed that the tunicamycin+PERK inhibitor treatment induced apoptosis in sphere-forming cells. The values shown represent the means ± SEM (*p < 0.05).

UPR branches balance pro-apoptosis and pro-survival signaling under ER stress. ER stress sensor inhibitors may disturb the balance caused by the shift of cells to one side. Monolayer and sphere-forming cells were exposed to the inhibitors of ER stress sensors, GSK2606414 (a PERK inhibitor: PERKi) or 4μ8C (an IRE1α inhibitor: IRE1i), combined with tunicamycin. Flow cytometric analyses for PI/Annexin-V and cell cycle proportions were performed on treated monolayer and sphere-forming cells in order to detect apoptotic cells (Figure 4 and Supplementary Figure S2). In monolayer cells, PERKi and IRE1i both promoted the induction of apoptosis caused by tunicamycin; neither PERKi nor IRE1i alone induced apoptosis (Supplementary Figure S2). This result indicated that both inhibitors sensitized monolayer cells to ER stress-mediated apoptosis because of the blockade of UPR branches followed by a disturbance in the UPR balance. Tunicamycin did not induce apoptosis in sphere-forming cells; the proportions of apoptotic cells detected by PI/Annexin-V and cell cycle proportion assays were only approximately 1-2%. These proportions in sphere-forming cells were increased to approximately 15% by the tunicamycin+PERKi treatment, but not by the tunicamycin+IRE1i treatment (Figure 4A, 4B, and 4C). This result indicated that PERKi, but not IRE1i sensitized sphere-forming cells to tunicamycin-induced apoptosis. UPR in response to tunicamycin balanced the inactivated IRE1 branch and activated PERK branch in sphere-forming cells. Therefore, the inhibition of the PERK branch disturbed pro-survival signaling, which was activated by tunicamycin, followed by a preferential shift to apoptotic signaling.

### Different UPR to cisplatin-mediated ER stress in cancer stem-like cells

**Figure 5:**
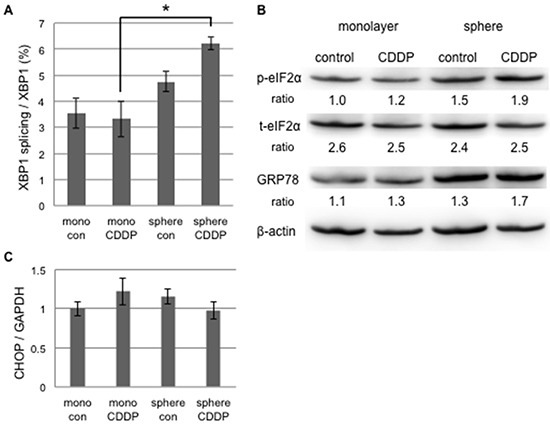
UPR to cisplatin-induced ER stress differed between cancer stem-like and cancer cells Monolayer (mono) or sphere-forming (sphere) cells were untreated (control: con) or treated with 20 μM cisplatin (CDDP) for 72 hours, and live cells were fractioned. **A.** Total RNA was extracted for RT-PCR and the ratio of spliced XBP1 mRNA to total XBP1 mRNA was calculated by the comparative Ct method. The values shown represent the means ± SEM (*p < 0.05). **B.** Cell extracts were prepared for Western blotting of the indicated proteins, and representative blots are shown. The ratio indicated that each protein level was normalized by β-actin levels (loading control). The phosphorylation of eIF2α was negligibly increased by the treatment with 20 μM cisplatin. **C.** The relative expression of CHOP was calculated and normalized by GAPDH. No significant change was observed in CHOP expression by the treatment with 20 μM cisplatin. The values shown represent the means ± SEM.

Cisplatin is a key anti-cancer drug that induces DNA damage followed by p53-dependent apoptosis and the inhibition of cell replication, and is also an ER stress inducer, which is followed by ER stress-mediated apoptosis [24, 25]. On the other hand, cisplatin-resistant cancer cells including CSC have the ability to evade cisplatin-induced apoptosis [26, 27]. Since our results indicated that the sphere-forming cells of cervical cancer evaded ER stress-mediated apoptosis, we addressed differences in UPR in response to cisplatin between monolayer and sphere-forming cells. In order to exclude the possibility of apoptosis due to cisplatin-dependent DNA damage, we used 20 μM of cisplatin in the treatment of monolayer and sphere-forming cells because a previous study reported that the concentration of cisplatin did not change the expression level of Bax [28]. We confirmed that 20 μM of cisplatin did not induce apoptosis in monolayer or sphere-forming cells (Supplementary Figure S3). At the concentration of cisplatin used, we examined the activities of the IRE1 and PERK branches in monolayer and sphere-forming cells. In monolayer cells, neither XBP-1 splicing nor elF2α phosphorylation was changed by the treatment with 20 μM of cisplatin (Figure 5), indicating that UPR branches were not activated by cisplatin-induced ER stress at that concentration. In contrast, XBP-1 splicing was enhanced by the treatment with 20 μM of cisplatin in sphere-forming cells, but not monolayer cells, whereas elF2α was not influenced (Figure 5A and 5B). The expression of CHOP and GRP78 was not changed in either cell. These results indicated that the low concentration of cisplatin activated the IRE1 branch in sphere-forming cells, but failed to activate ER stress-mediated apoptotic signaling.

Cisplatin has been shown to up-regulate GRP78 and shift exposed cells to ER stress-mediated apoptosis at a concentration of more than 20 μM [24]. In the present study, we used a low concentration of cisplatin in order to detect differences in sensitivity to cisplatin-induced ER stress between monolayer and sphere-forming cells. IRE1α was very weakly activated in sphere-forming cells only, while PERK was not activated. Martins et al recently demonstrated that cisplatin failed to activate PERK or the phosphorylation of elF2α whereas oxaliplatin did [25]. Our results also suggest that the IRE1 branch is pivotal in the balance of UPR to cisplatin-mediated ER stress for deciding cellular fates. This branch may be more susceptible to the cisplatin treatment in sphere-forming cells than in monolayer cells.

### The IRE1 inhibitor sensitized cancer stem-like cells to cisplatin-induced ER stress-mediated apoptosis

**Figure 6:**
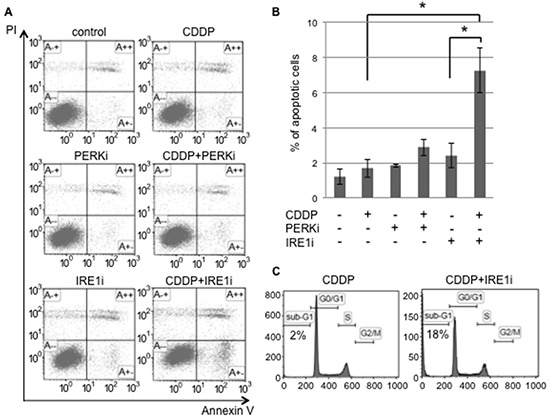
The IRE1α inhibitor induced cisplatin-mediated apoptosis in cancer stem-like cells Sphere-forming cells were untreated (control) or treated with 20 μM cisplatin (CDDP) and/or 1.5 μM GSK 2606414 (a PERK inhibitor: PERKi) or 7 μM 4μ8C (an IRE1α inhibitor: IRE1i) for 72 hours. **A.** After the treatment, cells were subjected to PI/Annexin-V staining and analyzed by flow cytometry. **B.** A quantitative analysis of PI-negative/Annexin-V-positive apoptotic cells showed that the proportion of apoptotic cells in sphere-forming cells was clearly increased by the IRE1α inhibitor combined with cisplatin treatment. The values shown represent the means ± SEM (*p < 0.05). **C.** After being treated, cells were fixed, stained with propidium iodide, and the sub-G1 population was calculated by flow cytometry. The cisplatin+IRE1α inhibitor treatment induced apoptosis in sphere-forming cells.

In order to confirm that the IRE1 branch plays a pivotal role in the UPR balance for sphere-forming cells against cisplatin, sphere-forming cells were exposed to either IRE1i (4μ8C) or PERKi (GSK2606414), with or without 20 μM of cisplatin, and the proportion of apoptotic cells was examined using flow cytometry for PI/Annexin-V (Figure 6). Cisplatin alone, PERKi alone, or cisplatin+PERKi failed to increase the proportion of apoptotic cells. In contrast, the proportion of apoptotic cells was 3 to 4-fold higher in cisplatin+IRE1i-treated cells than in control, cisplatin-treated, or IRE1i-treated cells (Figure 6B). As expected, the PERKi treatment did not influence the proportion of apoptotic cells regardless of the cisplatin treatment. We confirmed that cisplatin+IRE1i increased the proportion of sub-G1 phase cells in sphere-forming cells to approximately 18%, which was equivalent to that in tunicamycin-treated monolayer cells, as shown in Figure 2 (Figure 6C). These results clearly indicated that IRE1i sensitized sphere-forming cells to cisplatin-induced apoptosis.

**Figure 7:**
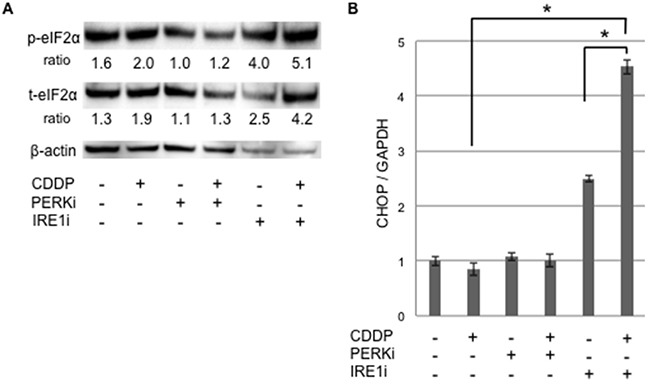
UPR production was increased in cancer stem-like cells treated with the IRE1α inhibitor combined with cisplatin Sphere-forming cells were untreated or treated with 20 μM cisplatin (CDDP) and/or 1.5 μM GSK 2606414 (a PERK inhibitor: PERKi) or 7 μM 4μ8C (an IRE1α inhibitor: IRE1i) for 72 hours, and live cells were fractioned. **A.** Cell extracts were prepared for Western blotting of the indicated proteins, and representative blots are shown. The ratio indicated that each protein level was normalized by β-actin levels (loading control). The phosphorylation of eIF2α was increased by the cisplatin+IRE1α inhibitor treatment. **B.** The relative expression of CHOP, normalized by GAPDH, was also increased by the cisplatin+IRE1α inhibitor treatment. The values shown represent the means ± SEM (*p < 0.05).

In order to determine whether apoptotic signaling was mediated by ER stress, we assessed the phosphorylation of elF2α and expression of CHOP in sphere-forming cells exposed to either IRE1i or PERKi with or without cisplatin (Figure 7). Western blotting for elF2α revealed that p-elF2α/β-actin was increased by cisplatin+IRE1i, and was decreased by PERKi as expected (Figure 7A). CHOP expression was increased significantly by cisplatin+IRE1i, but not by cisplatin alone, PERKi alone, IRE1i alone, or cisplatin+PERKi. The increase observed in the expression of CHOP paralleled the proportion of apoptotic cells shown in Figure 6B, indicating that cisplatin+IRE1i sensitized sphere-forming cells to ER stress-mediated apoptosis through the compensatory activation of elF2α signaling with the inhibition of IRE1.

UPR to ER stress is known to differ depending on the cell type, and the timing of UPR branch signaling determines cellular fates [16]. Our results demonstrated that sphere-forming cells showed different responses to ER stress inducers (tunicamycin and cisplatin), whereas these inducers alone failed to induce apoptosis in these cells. Regarding the tunicamycin treatment we used here, sphere-forming cells preferentially activated the PERK branch (PERK/p-elF2α) compared with monolayer cells. Therefore, the UPR balance may shift sphere-forming cells to cell survival rather than apoptosis. Our results indicate the potential of PERKi as an effective sensitizer to ER stress-mediated apoptosis for sphere-forming cells.

In contrast, the UPR balance in cisplatin-treated cells was markedly different from that in tunicamycin-treated cells. The UPR branch responsible for determining the balance from pro-apoptotic to pro-survival signaling was the IRE1 branch due to the lack of PERK activation by cisplatin [26]. The IRE1 branch was only activated in sphere-forming cells by the treatment with the low concentration of cisplatin (Figure 5A). The sensitivity of IRE1 to cisplatin-induced ER stress in sphere-forming cells may be higher than that in monolayer cells. Therefore, IRE1i sensitized sphere-forming cells to cisplatin-induced ER stress-mediated apoptosis. Notably, CHOP expression was increased in sphere-forming cells treated with cisplatin+IRE1i, but did not change with the cisplatin treatment. ElF2α was activated by the inhibition of the IRE1 branch, and this was followed by the induction of CHOP. The compensatory activation of the PERK branch occurred in sphere-forming cells, even with the cisplatin treatment. The UPR balance in sphere-forming cells may shift from the IRE1 to PERK branch in order to overcome the cisplatin-mediated suppression of PERK. These pro-apoptotic signals decided the fate of sphere-forming cells to apoptosis.

When combined with IRE1i, 20 μM of cisplatin shifted sphere-forming cells to ER stress-mediated apoptosis. The results of the present study suggest that inhibitors of ER stress sensors have the ability to sensitize CS-like cells to apoptosis. Several approaches for ER stress branches, including inhibitors of ER stress sensor, have been under development as therapies against cancer [17, 29]. However, the previous studies did not approach CSC, but cancer cells. The combination of cisplatin and IRE1i may be a novel approach to cisplatin-resistant cancer with the survival of CS-like cells through their evasion of apoptosis. Although further studies are needed in order to elucidate the mechanisms by which sphere-forming cells acquire resistance to ER stress, UPR to stress may differ between cancer cells and CS-like cells. Our results suggest that inhibitors of ER stress sensors combined with chemotherapeutics provide a novel insight into CSC-targeting therapy that shifts CS-like cell to ER stress-mediated apoptosis.

## MATERIALS AND METHODS

### Cell lines

SiHa (ATCC HTB-35), a human cervical squamous carcinoma cell line, was maintained in Dulbecco's Modified Eagle's Medium (Wako) supplemented with 10% fetal bovine serum (Gibco) and antibiotics (Wako) at 37°C in a humidified atmosphere with 5% CO_2_.

### Isolation of sphere-forming cells

Cells were cultured in DMEM/F-12 (Gibco) supplemented with 0.4% bovine serum albumin (Sigma-Aldrich), 20 ng/mL epidermal growth factor (Invitrogen), and 10 ng/mL basic fibroblast growth factor (Invitrogen) in ultra-low attachment plates at 37°C in a humidified atmosphere with 5% CO_2_. Floating spheres were collected by slow centrifugation and dissociated into single cells by 0.25% trypsin/EDTA (Wako).

### Treatment with tunicamycin / cisplatin and ER stress sensor inhibitors

Tunicamycin, cisplatin, GSK2606414, and 4μ8C were obtained from Wako. Cells were incubated in DMEM + 10% FBS + antibiotics in 10-cm normal plates at a density of 5×10^6^ cells/plate for monolayer cells or in DMEM/F-12 + 0.4% BSA + 20 ng/mL EGF + 10 ng/mL FGF in 10-cm ultra-low attachment plates at the same density for sphere-forming cells. After a 3-hour incubation, cells were treated with or without TM 0.03 μM/cisplatin 20 μM, GSK2606414 1.5 μM/4μ8C 7 μM for 72 hours.

### Surface marker analyses and ALDH activity of sphere-forming cells

After dissociating into single cells, sphere-forming cells were labeled with an APC-conjugated anti-human CD49f antibody (Miltenyi Biotec) or PE-conjugated anti-human CD44 antibody (Miltenyi Biotec) at 4°C for 10 minutes in the dark, and this was followed by washing with PBS containing 0.5% BSA. Isotype control samples were treated in an identical manner with an APC-labeled rat IgG2a antibody or PE-labeled mouse IgG1 antibody, respectively. Cell pellets were resuspended for an analysis by flow cytometry.

In order to identify the cell population with high ALDH enzymatic activity, an ALDEFLUOR^TM^ Kit (STEMCELL) was used as recommended by the manufacturer.

### Apoptotic analyses

Apoptosis was measured by flow cytometry using an Annexin-V-FITC Apoptosis Detection Kit (Abcam). Cells were treated with drugs for 72 hours, floating and attached cells were both collected, washed with PBS twice, and resuspended in buffer containing Annexin-V and PI according to the manufacturer's instructions. The proportions of PI-negative/Annexin-V-positive cells were examined.

A cell cycle analysis was performed as an alternative method to assess apoptosis. Cells were washed with PBS twice, fixed in 70% ethanol (Wako) at 4°C for 2 hours, washed twice again, resuspended in PBS containing 250 μg/mL of RNase A (Sigma-Aldrich) at 37°C for 20 min, and then incubated with 25 μg/mL propidium iodide (Sigma-Aldrich) at 4°C for 30 min in the dark.

All samples were acquired in a FACSCalibur system, and analyzed using Kaluza software.

### Western blotting

A phospho-eIF2α antibody and eIF2α antibody were obtained from Cell Signaling Technology, and an anti-GRP78 BiP antibody and anti-beta actin antibody were from Abcam.

After cells had been collected and dissociated with 0.25% trypsin/EDTA, dead cells were removed using a dead cell removal kit (Miltenyi).

The same amount of protein from whole cell lysates was separated using Tris-glycine gels, and transferred to nitrocellulose membranes. Membranes were blocked at room temperature for 1 hour in 5% milk/TBS-T, and this was followed by an incubation with primary antibodies diluted in 5% milk/TBS-T or 5% BSA/TBS-T (for P-eIF2α antibody) at 4°C overnight. After washing with TBS-T, HRP-conjugated secondary antibodies (DAKO) in 5% milk/TBS-T were incubated at room temperature for 1 hour. Blots were developed using Immobilon Western Chemiluminescent HRP Substrate (MILLIPORE) according to the manufacturer's instructions.

### RT-quantitative PCR

Total RNA was extracted from live cells using a Blood/Cultured Cell Total RNA Mini Kit (FAVORGEN), followed by reverse transcription. cDNA was amplified for 40 cycles in a Light Cycler 480 (Roche). The expression of CHOP was normalized using GAPDH mRNA as an internal standard, and splicing of XBP1 (XBP1s/XBP1t) was calculated by the comparative Ct method.

The primer pairs used were as follows; human total XBP1 (XBP1t) *5′-GGCATCCTGGCTTGCCTCCA-3′ and 5′-GCCCCCTCAGCAGGTGTTCC-3′*, human spliced XBP1 (XBP1s) *5′-CGCTTGGGGATGGATGCCCTG-3′* and *5′-CCTGCACCTGCTGCGGACT-3′*, human CHOP *5′-GGAGCATCAGTCCCCCACTT-3′* and *5′-TGTGGGA TTGAGGGTCACATC-3′*, and human GAPDH *5′-GAAA GGTGAAGGTCGGAGTC-3′* and *5′-GAAGATGGTGA TGGGATTTC-3′*.

### Statistical analysis

Data are presented as means ± SEM. Statistical analyses were carried out using the Student's *t-*test or Dunnett's analysis using JMP software. A value of p < 0.05 was considered significant. Asterisks indicate comparisons with significant differences (p < 0.05).

## SUPPLEMENTARY FIGURES


