# Supplementary material for: Inhibition of endoplasmic reticulum (ER) stress sensors sensitizes cancer stem-like cells to ER stress-mediated apoptosis
Source: Oncotarget. 2016 Jun 17;7(32):51854–64. doi: 10.18632/oncotarget.10126 (PMC5239519; doi:10.18632/oncotarget.10126)
Supplement: Supplementary file 1 [file oncotarget-07-51854-s001.pdf]

Inhibition of endoplasmic reticulum (ER) stress sensors sensitizes cancer stem-like cells to ER stress-mediated apoptosis

SUPPLEMENTARY FIGURES

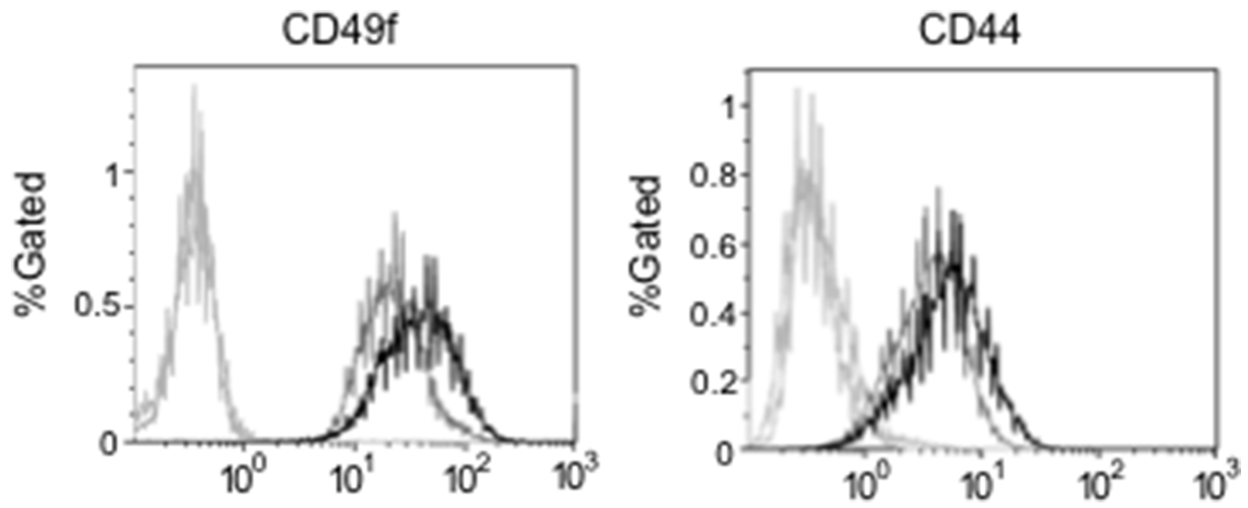

Supplementary Figure S1: The expression of CD44 and CD49f in sphere-forming cells derived from SiHa cells was slightly stronger than that in monolayer cells. Monolayer (gray) or sphere-forming cells (black) were labeled with APC-conjugated anti-human CD49f antibody or PE-conjugated anti-human CD44 antibody, and they were analyzed by flow cytometry. Isotype control samples are in light gray. The expression of CD44 and CD49f in sphere-forming cells was slightly stronger than that in monolayer cells.

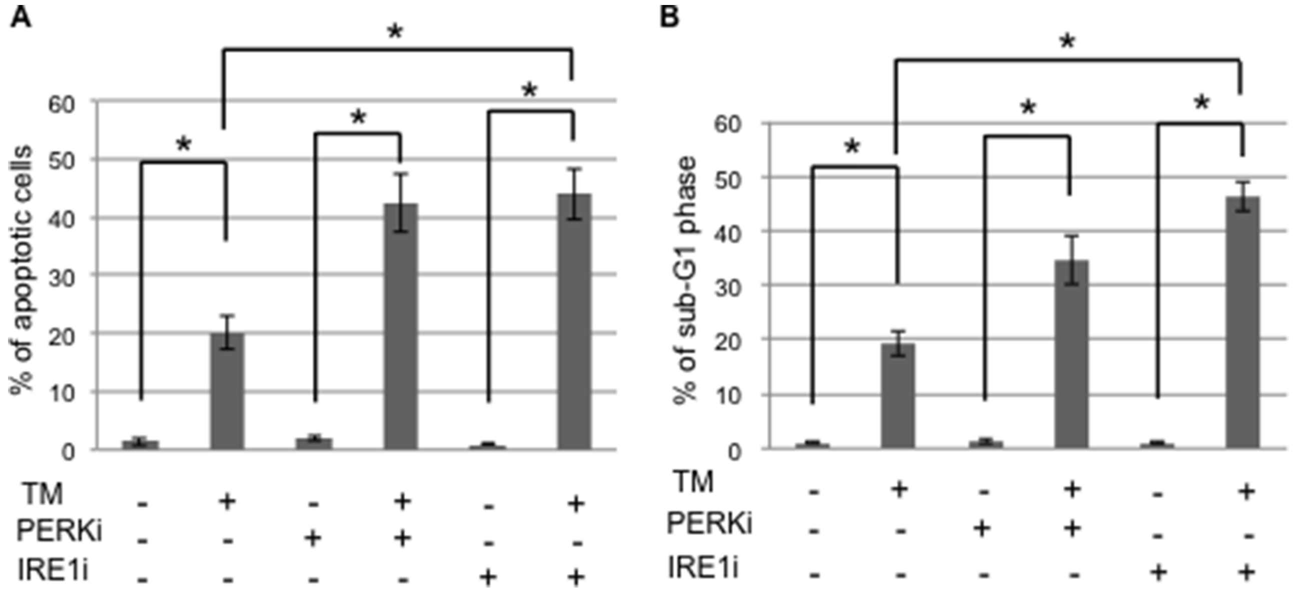

Supplementary Figure S2: ER stress sensor inhibitors induced tunicamycin-mediated apoptosis in cancer cells. Monolayer cells were untreated or treated with 0.03 µM tunicamycin (TM) and/or 1.5 µM GSK 2606414 (a PERK inhibitor: PERKi) or 7 µM 4µ8C (an IRE1α inhibitor: IRE1i) for 72 hours. **A.** After the treatment, cells were subjected to PI/Annexin-V-staining and analyzed by flow cytometry. A quantitative analysis of PI-negative/Annexin-V-positive apoptotic cells showed that the proportion of apoptotic cells in sphere-forming cells was clearly increased by the ER sensor inhibitor combined with tunicamycin treatment. The values shown represent the means ± SEM (*$p < 0.05$). **B.** Cells were fixed and stained with propidium iodide for a flow cytometry assay. A quantitative analysis of sub-G1 region (M1) cells showed that the ER sensor inhibitor combined with tunicamycin treatment induced apoptosis in sphere-forming cells. The values shown represent the means ± SEM (*$p < 0.05$).

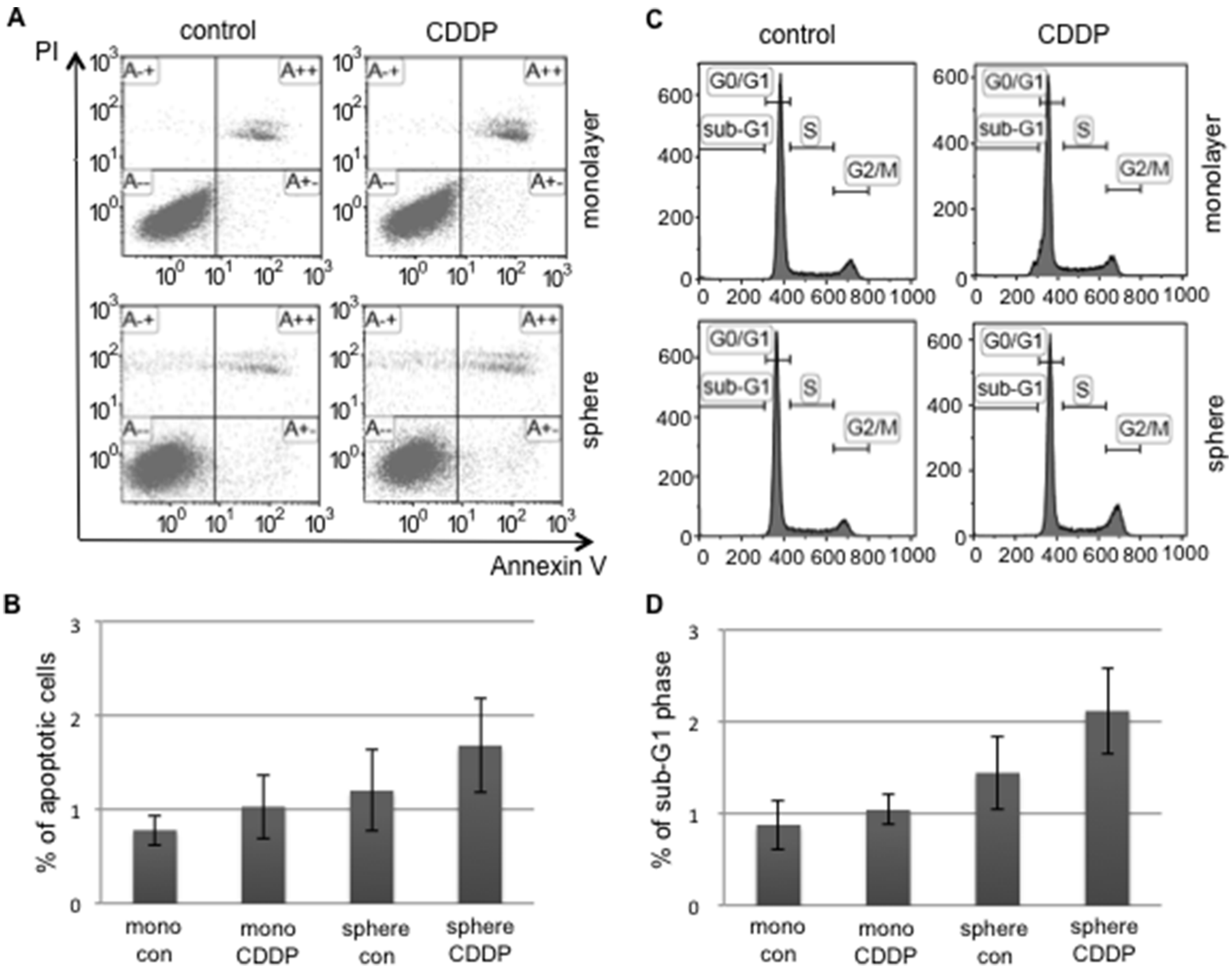

Supplementary Figure S3: Cisplatin-induced apoptosis occurred neither in monolayer cells nor in sphere-forming cells. A. Monolayer (mono) or sphere-forming (sphere) cells were untreated (control: con, left) or treated with 20 µM cisplatin (CDDP, right) for 72 hours, and then subjected to PI/Annexin-V staining and analyzed by flow cytometry. **B.** A quantitative analysis of PI-negative/ Annexin-V-positive apoptotic cells showed that cisplatin-induced apoptosis occurred neither in monolayer cells nor in sphere-forming cells. The values shown represent the means ± SEM. **C.** Monolayer or sphere-forming cells were untreated (left) or treated with 20 µM cisplatin (right) for 72 hours, then fixed and stained with propidium iodide for a flow cytometry assay. **D.** A quantitative analysis of sub-G1 region (M1) cells showed that cisplatin-induced apoptosis occurred neither in monolayer cells nor in sphere-forming cells. The values shown represent the means ± SEM.
